# Metformin-Associated Lactic Acidosis: A Case Report and Review

**DOI:** 10.7759/cureus.24220

**Published:** 2022-04-17

**Authors:** Shoaib Ashraf, Prakash Upreti, Sunita Karki, Muhammad Khan, Rabih Nasr

**Affiliations:** 1 Internal Medicine, BronxCare Health System/Icahn School of Medicine at Mount Sinai, Bronx, USA; 2 Internal Medicine, BronxCare Health System, Bronx, USA; 3 Nephrology, BronxCare Health System, Bronx, USA

**Keywords:** side effects, metformin, lactic acidosis, acute renal failure, diabetes mellitus

## Abstract

Metformin is widely prescribed as the first-line medication for type II diabetes mellitus. While the gastrointestinal side effects of metformin such as nausea, vomiting, diarrhea, and heartburn are quite common, one dangerous side effect of metformin, lactic acidosis, is extensively discussed yet rarely reported. Here, we discuss a 53-year-old female with type II diabetes mellitus who presented to an emergency department (ED) with chief complaints of dizziness and lightheadedness. The patient had chronic kidney disease (CKD) with a baseline estimated glomerular filtration rate (eGFR) of 45 mL/minute/1.73 m^2^. Initial laboratory results showed acute kidney injury (AKI) with hyperkalemia and lactic acidosis of 20 mmol/L. The patient was admitted to the ICU requiring emergent dialysis. Later, she was diagnosed with metformin-associated lactic acidosis (MALA). Her AKI and lactic acidosis subsequently improved. Metformin-associated lactic acidosis (MALA) is a rare but serious side effect of metformin. It is primarily reported in patients with chronic renal failure; therefore, it should be used with caution in these patients. Renal replacement therapy (RRT) is the critical management option for patients with MALA. Because of this, physicians prescribing metformin should carefully monitor all patients and assess the risk of developing severe side effects.

## Introduction

Metformin is a standard first-line drug to treat patients with type II diabetes mellitus [1]. Metformin is considered a harmless drug, but like any other drug, it has some side effects. It should not be used in patients with a high risk of lactic acidosis, such as those with old age, acute infection, progressively worsening renal and hepatic function, and circulatory problems such as congestive heart failure (CHF) [2]. Metformin-associated lactic acidosis (MALA) is a rare but serious adverse effect of metformin. Here, we discuss a 53-year-old female with type II diabetes mellitus on metformin who presented to the emergency department (ED) with chief complaints of dizziness and lightheadedness and was later diagnosed with MALA.

## Case presentation

We present a 53-year-old African American female who was brought to the emergency department (ED) with chief complaints of dizziness and lightheadedness since the morning of presentation associated with generalized weakness, fatigue, and shortness of breath a few days before presentation. She is a resident of a nursing home. Upon the arrival of emergency medical services at her nursing facility, her blood sugar was 34 mg/dL, and she was given dextrose. The patient was on metformin 850 mg twice a day for her diabetes mellitus. There was no recent change in medication dose or regimen, and she took the medication the previous night. She denied any chest pain, headache, blurry vision, syncope, new onset of weakness of any body part, abdominal pain, or nausea.

The patient has medical comorbidities of type II diabetes mellitus on metformin with the most recent hemoglobin A1c of 6.9%, hypertension, cerebrovascular accident, chronic kidney disease (CKD) stage 3a with baseline estimated glomerular filtration rate (eGFR) of 45 mL/minute/1.73 m^2^, and acquired thrombotic thrombocytopenic purpura on maintenance rituximab.

When she arrived in the ED, she was tachypneic and diaphoretic. Her initial vitals revealed a blood pressure of 109/52 mmHg, pulse of 121 bpm, respiratory rate of 25 breaths/minute, and saturating 98% on 2 L nasal cannula. Her finger stick blood glucose was 91 mg/dL. She stated that her dizziness and lightheadedness have resolved. Systemic examination was significant for tachycardia, tachypnea, mild bilateral lower extremity edema, and weakness of the left side, residual from her previous stroke.

Her initial blood laboratory work was significant for severe acute kidney injury (AKI) with a creatinine of 6.6 mg/dL with a baseline of 1.4 mg/dL, eGFR of 8.51 mL/minute/1.73 m^2^, potassium of 6.7 mmol/L, and blood gas showing severe anion gap (AGAP) metabolic acidosis with a lactate of 20 mmol/L (Table 1). Other significant laboratory results include serum acetaminophen level of <0.3 mg/dL (normal range: 3-10 mg/dL), hemoglobin A1c of 4.6%, insulin level of 9.7 uIU/mL (optimal: ≤19.6 uIU/mL), serum creatine kinase of 21 unit/L (normal range: 20-200 unit/L), and ethanol level of <10 mg/dL. The intensive care unit (ICU) was consulted for severe AKI, hyperkalemia, and possible sepsis, and nephrology was consulted for emergent dialysis. The patient was started on broad-spectrum antibiotics, and immediate central venous access was obtained for hemodialysis. She received hemodialysis in the ED and was transferred to the ICU for further management. In the ICU, her AKI and other blood parameters improved and did not require further dialysis. The patient was later transferred to the medical floor.

**Table 1:** Abnormal blood test trend

Date	pH	HCO_3_ (mmol/L)	Creatinine (mg/dL)	Potassium (mmol/L)	AGAP (mmol/L)	Lactic acid (mmol/L)
5/10/21	7.14	6	6.6	6.7	42	20
5/10/21	7.27	11	5.9	4.9	37	12.1
5/11/21	7.42	20	4.1	4.4	29	10.5
5/11/21	7.40		4.4	3.4	24	7.7
5/12/21	7.41	26	3.7	3.6	16	3.3
5/13/21			3.1	3.7	15	1.7
5/14/21			2.4		13	
5/15/21			1.9		11	1.6
5/16/21			1.5			
5/20/21			1.3			

Blood culture on admission revealed polymicrobial bacteremia including vancomycin-resistant* Enterococcus faecium*,* Enterococcus faecalis*, and *Staphylococcus epidermidis*, which raised suspicion for contaminated specimens; repeat blood cultures, urine culture, and pneumonia workup came back negative. The patient remains afebrile and never requires pressors or hemodynamic support during the clinical course, lowering the suspicion of underlying sepsis. Broad-spectrum antibiotics were later discontinued after a negative septic workup. The patient's renal function eventually improved with a creatinine of 1.4 mg/dL on day 8 of admission, and she was finally discharged back to the nursing home.

## Discussion

Metformin is a very widely prescribed antidiabetic medication that belongs to the biguanide class of oral hypoglycemic agents. It decreases glucose production in the liver and increases insulin activity in specific organs, particularly muscles and fat. Like other biguanides, metformin is also known to increase plasma lactate levels [1]. The oral bioavailability of metformin is 50%-60% [2]. Metformin is eliminated in unchanged form by the kidneys. Renal failure can cause metformin to pile up, which increases the risk of toxicity in these patients. Physicians should discontinue metformin when eGFR is <30 mL/minute/1.73 m^2^. According to the current recommendations, it is used with extreme caution in the ranges of 30-50 mL/minute/1.73 m^2^ [3].

Common side effects include abdominal discomfort, nausea, vomiting, anorexia, diarrhea, and a metallic taste in the mouth. Less common side effects include hemolytic anemia, decreased absorption of folic acid and vitamin B12, acute hepatitis, and lactic acidosis. It should not be used in patients with a high risk of lactic acidosis, such as very old patients, patients with hepatic and renal dysfunction, and patients with circulatory problems such as congestive heart failure [4].

MALA is clinically defined as metabolic acidosis (pH < 7.35) associated with serum lactate of >5 mmol/L in patients treated with metformin. The incidence of MALA is approximately 19 per 100,000 patient-year exposures to the drug [5]. Lactic acidosis due to metformin can be attributed when the plasma metformin level is more than 5 ug/mL. Such high levels (therapeutic plasma metformin level < 2 ug/mL) are primarily observed in patients with chronic or newly diagnosed renal failure (i.e., decreased clearance of metformin) and hepatic dysfunction (i.e., reduced clearance of lactate) complicated by lactate overproduction (i.e., CHF, anoxia, sepsis, and impaired perfusion of tissues) [4]. Our patient was on metformin 850 mg twice a day. There was no change in her medication dose or regimen. She had chronic kidney disease with a borderline eGFR of 45 mL/minute/1.73 m^2^ and baseline creatinine of 1.4 mg/dL. On her presentation, her creatinine was 6.6 mg/dL. Our patient had metabolic acidosis at pH 7.14 with a lactate level of 20 mmol/L and was on metformin, which meets the criteria for MALA.

Patients with MALA present with nonspecific symptoms such as vomiting and diarrhea, abdominal pain, watery diarrhea, lethargy, somnolence, and thirst. Other reported presentations are hypotension, hypoglycemia, hypothermia, and severe respiratory dysfunction necessitating mechanical ventilation [6]. Cardiac arrhythmias, including bradycardia, asystole, ventricular fibrillation, and multifocal ventricular extrasystoles, can occur in these patients mainly due to acidemia [7]. Lactic acidosis, along with gastrointestinal symptoms, can be misdiagnosed as mesenteric ischemia. It can lead to increased mortality due to misdiagnosis [8]. It could be one of the reasons for the underdiagnosis of MALA in the real world.

MALA treatment is usually supportive therapy with hemodynamic stability, but severe cases might require mechanical ventilation, vasopressors, and renal replacement therapies (RRT) [1]. It eliminates blood metformin and improves acidosis. RRT is mainly used in patients with severe metabolic acidosis and critically ill patients with significant comorbidities. It is also used in patients with severe renal insufficiency and patients with inadequate response to supportive treatment. Hemodialysis is performed using bicarbonate buffer to correct metabolic acidosis. Continuous venovenous hemodialysis (CVVHD) is also being used in patients with metformin overdose. However, it is found that the clearance of metformin is lesser than in conventional hemodialysis. Hence, CVVHD should only be considered in hemodynamically unstable patients who do not tolerate conventional HD [1]. In one case series comprising 42 patients, it was reported that the total duration of hemodialysis of 15 hours resulted in the return of metformin level to the therapeutic range [9]. In our patient, conventional hemodialysis was performed, which resulted in improvement of acidosis and renal recovery.

Mortality in patients with metformin-induced lactic acidosis ranges from 25% to 49% in different studies [1,9-11]. Lactate and plasma metformin levels are not good predictors of mortality [11].

## Conclusions

Metformin-associated lactic acidosis is an uncommon side effect of metformin. It is primarily reported in patients with chronic renal failure; therefore, metformin should be used with caution in these patients. Patients with CKD on metformin should be monitored for worsening renal function and followed closely. Metformin should be held when eGFR falls to less than 30 mL/minute/1.73 m^2^. Patients presenting to the emergency department with metformin-associated lactic acidosis can have nonspecific symptoms. These patients require emergent dialysis with ICU level of care, and their blood parameters such as lactic acid level, anion gap (AGAP), and renal function should be closely monitored.
